# The relationship between physical activity, apolipoprotein E ε4 carriage, and brain health

**DOI:** 10.1186/s13195-020-00608-3

**Published:** 2020-04-24

**Authors:** Jaisalmer de Frutos-Lucas, Pablo Cuesta, David López-Sanz, África Peral-Suárez, Esther Cuadrado-Soto, Federico Ramírez-Toraño, Belinda M. Brown, Juan M. Serrano, Simon M. Laws, Inmaculada C. Rodríguez-Rojo, Juan Verdejo-Román, Ricardo Bruña, Maria L. Delgado-Losada, Ana Barabash, Ana M. López-Sobaler, Ramón López-Higes, Alberto Marcos, Fernando Maestú

**Affiliations:** 1 Biological and Health Psychology Department, School of Psychology, Universidad Autonoma de Madrid, 28049 Madrid, Spain; 2 Laboratory of Cognitive and Computational Neuroscience (UCM-UPM), Center for biomedical Technology, Parque Científico y Tecnológico de la UPM, Crta. M40, Km. 38, 28223 Pozuelo de Alarcón, Madrid Spain; 3 Collaborative Genomics Group, School of Medical and Health Sciences, Edith Cowan University, Joondalup, Western Australia 6027 Australia; 4 Department of Industrial Engineering & IUNE, Universidad de La Laguna, 38200 San Cristobal de la Laguna, Tenerife Spain; 5 Department of Psychobiology and Methodology in Behavioral Sciences, School of Education, Universidad Complutense de Madrid, 28040 Madrid, Spain; 6 Departamento de Nutricion y Ciencia de los Alimentos, Facultad de Farmacia, Universidad Complutense de Madrid, 28040 Madrid, Spain; 7 Experimental Psychology Department, School of Psychology, Universidad Complutense de Madrid, 28223 Pozuelo de Alarcon, Spain; 8 Discipline of Exercise Science, College of Science, Health, Engineering and Education, Murdoch University, Murdoch, Western Australia 6150 Australia; 9 School of Pharmacy and Biomedical Sciences, Faculty of Health Sciences, Curtin Health Innovation Research Institute, Curtin University, Bentley, Western Australia 6102 Australia; 10 Centro Universitario Villanueva, Facultad de Psicología, 28034 Madrid, Spain; 11 Mind, Brain and Behavior Research Center (CIMCYC), Universidad de Granada, 18071 Granada, Spain; 12 Networking Research Center on Bioengineering, Biomaterials and Nanomedicine (CIBER-BBN), 28029 Madrid, Spain; 13 Endocrinology and Nutrition Department, Hospital Clinico San Carlos and Instituto de Investigación Sanitaria del Hospital Clínico San Carlos, 28040 Madrid, Spain; 14 Centro de Investigación Biomédica en Red de Diabetes y Enfermedades Metabólicas Asociadas, 28040 Madrid, Spain; 15 Neurology Department, Hospital Clinico San Carlos and Instituto de Investigación Sanitaria del Hospital Clínico San Carlos, 28040 Madrid, Spain

**Keywords:** Physical activity, *APOE*, MEG, Synaptic function, Alzheimer’s disease, Temporal lobe

## Abstract

**Background:**

Neuronal hyperexcitability and hypersynchrony have been described as key features of neurophysiological dysfunctions in the Alzheimer’s disease (AD) continuum. Conversely, physical activity (PA) has been associated with improved brain health and reduced AD risk. However, there is controversy regarding whether AD genetic risk (in terms of *APOE* ε4 carriage) modulates these relationships. The utilization of multiple outcome measures within one sample may strengthen our understanding of this complex phenomenon.

**Method:**

The relationship between PA and functional connectivity (FC) was examined in a sample of 107 healthy older adults using magnetoencephalography. Additionally, we explored whether ε4 carriage modulates this association. The correlation between FC and brain structural integrity, cognition, and mood was also investigated.

**Results:**

A relationship between higher PA and decreased FC (hyposynchrony) in the left temporal lobe was observed among all individuals (across the whole sample, in ε4 carriers, and in ε4 non-carriers), but its effects manifest differently according to genetic risk. In ε4 carriers, we report an association between this region-specific FC profile and preserved brain structure (greater gray matter volumes and higher integrity of white matter tracts). In this group, decreased FC also correlated with reduced anxiety levels. In ε4 non-carriers, this profile is associated with improved cognition (working and episodic memory).

**Conclusions:**

PA could mitigate the increase in FC (hypersynchronization) that characterizes preclinical AD, being beneficial for all individuals, especially ε4 carriers.

## Background

Physical activity (PA) has been persistently referred to as the twenty-first century panacea. In both clinical and non-clinical populations, PA is related to improvements in sleep quality, mood, cognitive performance, and perceived quality of life [1]. In addition, PA is associated with marked decreases in the risk of a broad spectrum of diseases, including diabetes mellitus, cancer, and dementia [1]. In the specific case of Alzheimer’s disease (AD; the most common form of dementia), PA has been found to reduce incidence, AD-associated neuropathological burden, and cognitive decline [2–4].

The major genetic risk factor for sporadic AD, namely the apolipoprotein E (*APOE*) ε4 allele, is present in 60–80% of AD cases and is linked to a 3.2-fold increased AD risk in heterozygosis and up to 14.9-fold increased AD risk in homozygosis [5]. While most studies claim that PA-protective effects mainly manifest among ε4 carriers [6, 7], others identified this same effect exists only in non-carriers [8, 9], while other studies report benefits regardless of *APOE* ε4 allele carriage [10, 11]. This inconsistent literature is likely due to the use of varying study designs and outcome measures (i.e., hippocampal volume, short-term memory, or rate of AD conversion). To further understand the modulating effect of AD genetic risk on the relationship between PA and brain health, a more detailed investigation using varied outcome measures is warranted.

Based on the inconsistencies in previous literature, the current study will first investigate the relationship between PA and synaptic activity in cognitively healthy older adults. Synaptic activity will be captured employing magnetoencephalography and analyzed under the framework of network synchronization. It is believed that the flow of information between different brain regions is sustained by synchronous changes in the frequency, pattern, or strength of their oscillatory activity [12]. Early loss of inhibitory neurons in preclinical AD leads to a state of increased hyperexcitability and hypersynchrony [13–15], which has been found to augment amyloid release and produce neurotoxic effects [16, 17]. We hypothesize that PA exerts a neuroprotective effect that will be associated with reduced network synchrony in both groups, in opposition to the state of synaptic hyperexcitability that characterizes preclinical and prodromal AD [18–22]. Then, once we have identified the functional connectivity (FC) patterns that are influenced by PA level, we will explore if there are any associations between these FC patterns and structural integrity (gray and white matter), cognition, and mood. Additionally, we will examine if *APOE* ε4 allele carriage modulates these relationships.

## Methods and materials

### Participants

A total of 262 individuals participated in a study aimed to characterize the neurophysiological features of healthy aging. Participants were recruited from local hospitals and through several dissemination talks, and a team of expert neuropsychologists assessed that they met inclusion criteria. A detailed list of exclusion criteria can be found in [23]. The procedure was performed following current guidelines and regulations, and the study was approved by the Hospital Universitario San Carlos Ethics Committee. Every participant signed an informed consent.

We included participants who had available data regarding our main variables of interest (*n* = 158; Mini-Mental State Examination (MMSE) score, genetic information, and validated magnetic resonance imaging (MRI), MEG, and actigraphy data). We then excluded anyone with an MMSE score less than 26 (*n* = 5), aged less than 50 years (*n* = 8), and participants carrying less frequent APOE genotypes (ε2ε3, *n =* 11; ε2ε4, *n =* 1; ε4ε4, *n =* 7; there were no ε2ε2 homozygotes in the original cohort). We focused on the comparison between individuals at standard genetic risk for AD (ε3ε3; hereafter non-carriers) and individuals at increased genetic risk for AD in heterozygosis (ε3ε4; hereafter; carriers) since sample sizes were insufficient to separately study the effects ε2 carriage (linked to reduced risk of AD but increased risk of type III hyperlipoproteinemia [24]) and ε4 carriage in homozygosis. Nevertheless, excluded genotypes are known to alter molecular and cellular dynamics [24, 25], which could potentially interfere with the neurophysiological response to exercise, and therefore, we decided not to group together all ε4-carrying (ε2ε4, ε3ε4, ε4ε4) and all ε4-non-carrying (ε2ε2, ε2ε3, ε3ε3) genotypes. Among the remaining 127 participants, there were 36 *APOE* ε4 carriers and 91 non-carriers. We carefully selected 33 *APOE* ε4 carriers and 74 non-carriers so that both subsamples would match in PA levels (TPA and MVPA), age, sex, educational level, MMSE, and body mass index. There were two main reasons to match the sample according to all these relevant variables instead of using them as covariates in subsequent analyses. First, including several covariates in a cluster-based permutation test could have introduced a methodological pitfall in the permutation procedure. Second, using covariates only controls for linear influences on the data, dismissing any other possible non-linear confound.

The final sample was composed of 107 healthy older adults, aged 50–82 years. A detailed list of the sample characteristics can be found in Table 1, including scores extracted from the neuropsychological tests: Geriatric Depression Scale [26], the anxiety subscale from the Goldberg Anxiety and Depression Inventory [27], and the Digit Span Forward, Digit Span Backward, and Logical Memory II (delayed recall, units, and gist) subscales from the Weschler Adult Intelligence Scale IV (WAIS-IV, [28]).

**Table 1:** Descriptive measures of the final sample

Variable	Whole sample	Carriers	Non-carriers	*p* value
Sex (M; F)	32; 75	8; 25	24; 50	0.49
Age	60.48 ± 8.10	59.36 ± 7.46	60.97 ± 8.36	0.39
Education	4.62 ± 0.68	4.61 ± 0.75	4.622 ± 0.63	0.86
MMSE	29.26 ± 0.84	29.33 ± 0.89	29.23 ± 0.82	0.42
BMI	25.03 ± 3.61	25.01 ± 3.82	25.04 ± 3.55	0.90
TPA	0.0125 ± 0.0117	0.0109 ± 0.0107	0.0133 ± 0.0122	0.48
MVPA	36.34 ± 21.12	33.12 ± 17.64	37.77 ± 22.47	0.54
Forward digits	5.95 ± 1.22	5.88 ± 1.22	5.99 + 1.23	0.75
Reverse digits	4.50 ± 1.36	4.67 ± 1.65	4.43 ± 1.22	0.55
Logical mem. units	18.79 ± 11.13	20.97 ± 11.87	17.86 ± 10.76	0.25
Logical mem. gist	15.74 ± 10.87	13.69 ± 8.14	16.63 ± 11.80	0.52
Anxiety	1.71 ± 0.84	1.86 ± 2.37	1.65 ± 2.05	0.84
Depression	1.24 ± 1.62	1.27 ± 1.44	1.23 ± 1.70	0.70
Total GM	545,939 ± 51,206	546,825 ± 66,891	545,538 ± 42,833	0.80
Hippocampus	7549 ± 834	7583 ± 954	7533 ± 780	0.81
Amygdala	2730 ± 470	2721 ± 573	2734 ± 420	0.51
Precuneus	16,337 ± 2002	16,403 ± 2491	16,306.83 ± 1757	0.87
Global FA	0.4355 ± 0.017362	0.4395 ± 0.01645	0.4336 ± 0.0176	0.11
Uncinate	0.4378 ± 0.0238	0.4387 ± 0.0232	0.4374 ± 0,0242	0.87
Parahippocampus	0.4355 ± 0.0173	0.4177 ± 0.0315	0.4145 ± 0.0289	0.71

Mean values ± standard deviation for all matching variables as well as variables used for correlation analyses. These include sex (where M stands for male and F for female); age (in years); education (in terms of educational level on a 0—illiterate—to 5—postsecondary education—scale); Mini-Mental State Examination (MMSE); body mass index (BMI); total physical activity (TPA, normalized by actigraphy wear time); daily average of moderate to vigorous physical activity (MVPA, in minutes); working memory (Forward and Reverse Digit Span—forward and reverse digits); episodic memory (Logical Memory II—delayed recall; for units and gist—logical mem. units and gist); anxiety (Goldberg’s test); depression (Geriatric Depression Scale); total gray matter volume (GM, in cubic millimeter); hippocampus, amygdala, and precuneus volumes (left plus right, in cubic millimeter); global fractional anisotropy (FA); and uncinate and parahippocampal fasciculus fractional anisotropy (left and right weighted arithmetic mean). Results are displayed for the whole sample and also for each subsample of interest (*APOE* ε4 carriers and non-carriers). *p* values for the Mann-Whitney and Fisher (sex) tests are also shown. No significant between-group differences arose across all comparisons

### Physical activity measurement

For PA measurement, we used the ActiGraph GT3X+ accelerometer (LLC, Pensacola, FL). Participants were requested to wear the accelerometers on their right hip for 7 complete days, taking them off only during water-based activities [29, 30]. For cleaning and processing the data, we used the ActiLife software (6.13.3) (LLC, Pensacola, FL). The validation criteria required each individual to wear the accelerometer during at least 3 weekdays and 1 weekend day for a minimum of 10 h per day [30]. We considered ≥ 60 min of continuous zeroes while allowing for up to 2 min of counts ≤ 100 counts as non-wear time [31]. To classify the PA, we categorized sedentary time as < 100 counts/min, light activity as 100–1951 counts/min, and moderate to vigorous physical activity (MVPA) as ≥ 1952 counts/min [32].

In this study, two different measures of PA were incorporated: Total Time In Freedson Bouts, which is a standardized measure of PA volumes (total PA, TPA), and daily average of MVPA. TPA was normalized by total wear time.

### APOE genotyping

As described in [23], we obtained genomic DNA from 10 ml blood samples in ethylenediaminetetraacetic acid (EDTA). Employing TaqMan assays on an Applied Biosystems 7500 Fast Real Time PCR machine (Applied Biosystems, Foster City, CA), single nucleotide polymorphisms (SNPs) rs7412 and rs429358 genotypes were determined. *APOE* genotype was established accordingly. In this study, only ε3ε3 and ε3ε4 individuals were considered.

### MRI acquisition and volumetric analyses

To generate the T1-weighted MRI images from each participant, a General Electric 1.5-T system was employed. We applied a high-resolution antenna and a homogenization PURE filter (Fast Spoiled Gradient Echo sequence, TR/TE/TI = 11.2/4.2/450 ms; flip angle 12°; 1 mm slice thickness, 256 × 256 matrix, and FOV 25 cm).

The resulting images were processed using the Freesurfer software (version 5.1.0) and its specialized tool for automated cortical parcellation and subcortical segmentation [33]. The measures that were included in further analyses were total gray matter, amygdala, precuneus, and hippocampus (in cubic millimeter). The volumes of bilateral structures were collapsed in order to obtain a single measure for each region.

### Diffusion tensor imaging

#### Data acquisition

The same General Electric 1.5-T magnetic resonance scanner was also used to collect diffusion-weighted images (DWI). The acquisition parameters for DWI were as follows: TE/TR 96.1/12,000 ms; NEX 3 for increasing the SNR; 2.4 mm slice thickness, 128 × 128 matrix, and 30.7 cm FOV yielding an isotropic voxel of 2.4 mm; 1 image with no diffusion sensitization (i.e., T2-weighted b0 images); and 25 DWI (*b* = 900 s/mm^2^). Data were recorded with a single shot echo planar imaging sequence.

#### Preprocessing

DWI images were processed following the procedure previously published in [34]. Probabilistic fiber tractography was run on the automated tool AutoPtx (https://fsl.fmrib.ox.ac.uk/fsl/fslwiki/AutoPtx) as reported in [34]. Two bilateral tracts were later used for correlation analyses: the uncinate and the parahippocampal fasciculi. In order to reduce the number of tests, the weighted arithmetic mean of left and right structures was used. Likewise, a measure of global fractional anisotropy (FA) was calculated averaging all 27 original tracts provided by the system.

### Magnetoencephalography

#### Data acquisition and signal preprocessing

MEG data was recorded using a 306-channel whole-head MEG system (Vectorview, ElektaNeuromag, Finland), placed in a magnetically shielded room at the Center for Biomedical Technology in Madrid, following the protocol described in [23].

Raw data were first submitted to the Maxfilter software to remove external noise [35]. Fieldtrip software [36] was used to automatically scan MEG data for artifacts, which were visually confirmed by an MEG expert. Artifact-free data were segmented in 4 s epochs. Then, MEG time series were filtered into delta (2–4 Hz), theta (4–8 Hz), alpha (8–12 Hz), and beta (12–30 Hz). This procedure has been reported in detail in [22].

#### Source reconstruction and connectivity analyses

We used a regular 1-cm grid in the Montreal Neurological Institute (MNI) template. The resulting model comprised 2459 sources distributed across the brain, which were transformed to each subject’s space following the methodology detailed in [22].

We used phase locking value (PLV) to calculate functional connectivity. The Automated Anatomical Labeling atlas (AAL, [37]) was applied to segment the source template with 2459 nodes excluding the cerebellum, basal ganglia, thalamus, and olfactory cortices. The resulting 78 regions of interest included 1202 nodes. Symmetrical whole-brain matrices of 1202 × 1202 nodes were obtained by averaging PLV values across trials for each participant and frequency band. Each node’s strength was computed by averaging its corresponding FC with the whole grid. Such averaging resulted in a source-reconstructed FC matrix of 1202 nodes by 4 frequency bands by 107 participants.

### Statistical analyses

#### Functional connectivity strength clustering

Network-based statistics (NBS) were carried out for each frequency band [38]. Clusters consisted of several spatially adjacent nodes that presented a significant partial correlation (age as covariate) between functional connectivity strength (FC-st) values and each PA variable (Spearman’s correlation, *p* < 0.01). To form a cluster, the correlation coefficients of all nodes within the cluster were required to have the same sign. Only clusters including at least 1% of the grid (i.e., a minimum of 12 nodes) were considered (i.e., minimum size condition). Spearman’s rho values were Fisher *Z*-transformed. Cluster-mass statistics were computed as the sum of all *Z* values corresponding with all nodes within each cluster. Moderation analyses were carried out to study the possible influence exerted by *APOE* genotype (ε4 carriers vs non-carriers) in the reported relationship between FC-st and TPA. We employed multiple regression analysis and calculated the increase in variance explained by our model after including the interaction term (*APOE*_by_TPA). This model first used TPA and *APOE* as predictors to linearly estimate FC-st in the significant cluster. Then, the interaction term was added (TPA_by_*APOE*). The *p* value for this interaction term is interpreted as the moderating effect significance.

#### Controlling for multiple comparisons

To control for multiple comparisons, the whole process was repeated 5000 times, shuffling the correspondence between FC-st and each PA measure across all participants. At each repetition, the maximum surrogate cluster’s statistic was kept creating a maximal null permutation distribution. For each main cluster, cluster-mass statistics in the original and the randomized datasets were compared. In NBS, *p* value represents the proportion of the permutation distribution with cluster-mass statistic values greater or equal than the cluster-mass statistic value of the original data. Only clusters which survived NBS (permutation *p* value < 0.05) were considered in further analyses. For each main cluster, FC-st values were averaged across all nodes to obtain cluster’s representative MEG markers.

#### Correlations between FC-st and markers of brain function and structure

These markers were used in subsequent correlation analyses with measures of specific AD signatures (the complete list is shown in Table 4). These were carried out taking the whole sample and following stratification of the cohort by *APOE* ε4 carriers and non-carriers. *p* values were corrected using false discovery rate (FDR) to account for multiple testing. All statistical analyses were carried out using MATLAB R2018b (Mathworks Inc).

#### Seed-based analyses

In order to examine whether the FC-st results were caused by global or between-region-specific effects, we performed corresponding seed analysis, using the previous clusters as seeds. The FC values assessed were the average FC between each node of the grid and corresponding cluster’s nodes. Then, a set of partial correlation (age as covariate) between these FC values and each PA variable (Spearman’s correlation, *p* < 0.01) were computed. Only clusters that did not overlap with the original seed cluster were reported in this study.

## Results

### Physical activity is associated with decreased temporal lobe FC-st in ε4 carriers and non-carriers

FC-st is computed as the average FC between each specific source and the rest of the network. Here, we examined whether any brain regions, henceforth referred to as clusters, had FC-st values that significantly correlated with PA levels, using age as a covariate. Significant clusters comprised brain regions that behave as functional units.

**Fig. 1:**
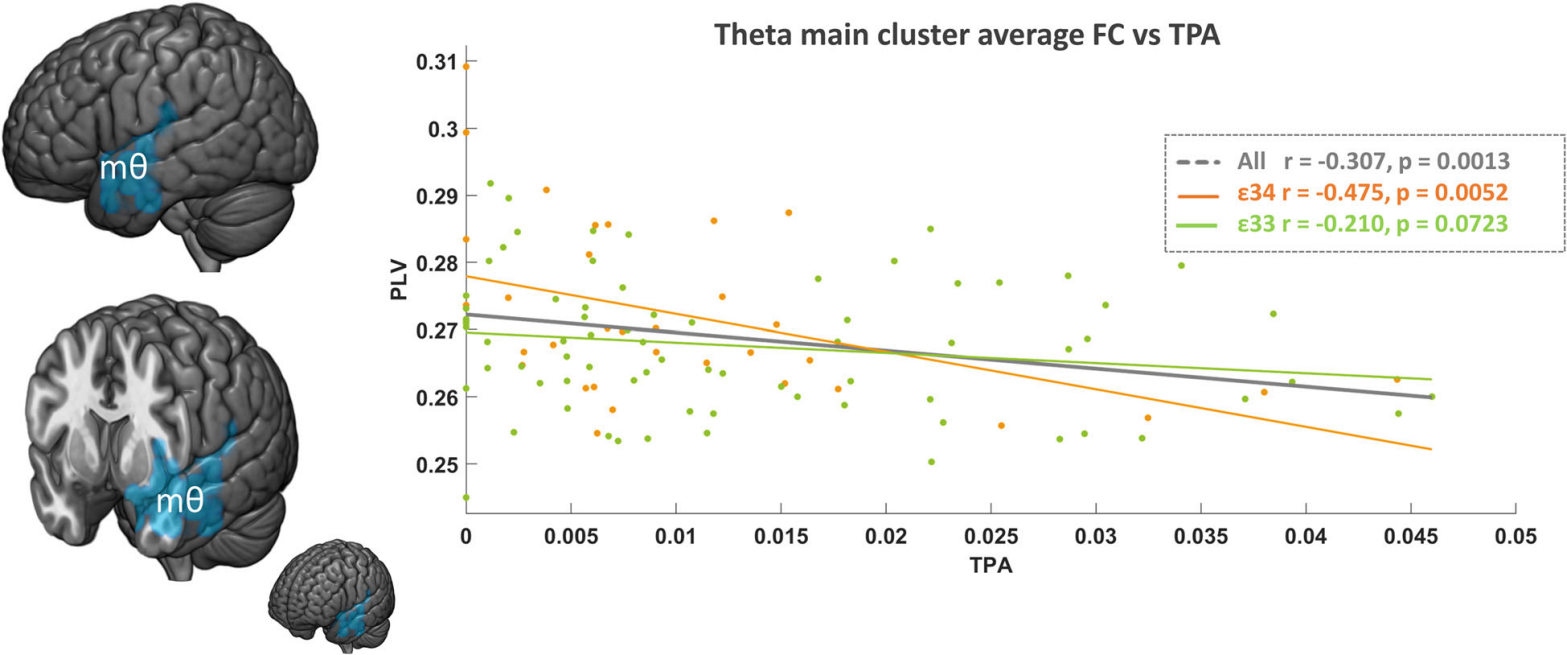
FC-st results in the theta band. In dark blue, marked as mθ, the brain region whose functional connectivity strength (FC-st) was found inversely correlated with total physical activity (TPA) is displayed. On the right, the scatter plot shows the correlation between mθFC-st and TPA computed with the whole sample (gray), *APOE* ε4 carriers (orange), and non-carriers (green)

**Fig. 2:**
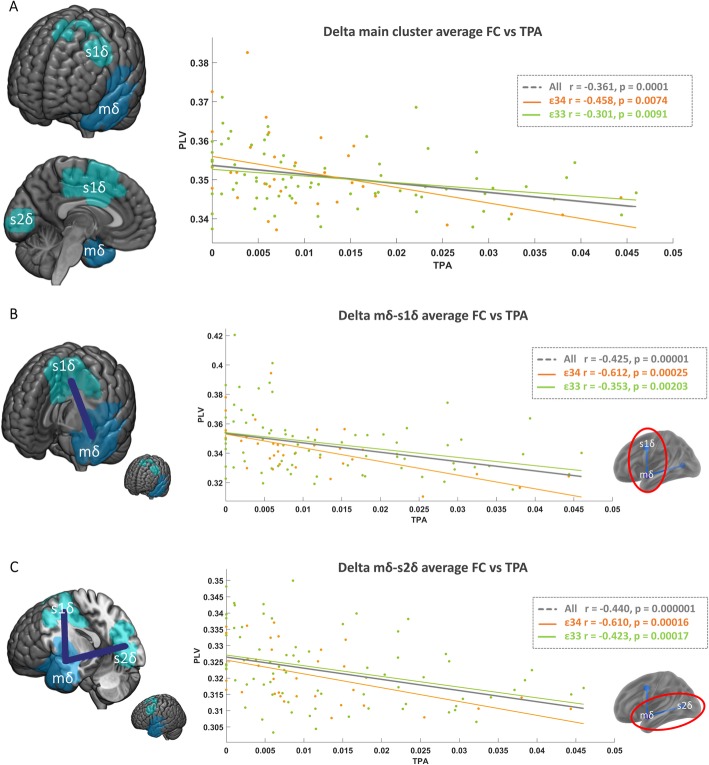
FC-st results in the delta band. **a** In dark blue, marked as mδ, the brain region whose functional connectivity strength (FC-st) was found inversely correlated with total physical activity (TPA) is displayed. In light blue, those regions, marked as s1δ and s2δ, whose FC with mδ was found to inversely correlate with TPA are depicted. On the right, the scatter plot shows the correlation between mδ FC-st and TPA computed with the whole sample (gray), *APOE* ε4 carriers (orange), and non-carriers (green). **b** Seed 1 results (s1δ). Purple line represents the significant FC link whose value is included in the correlation analysis. The correlation between mδ <−> s1δ FC and TPA is shown on the right. **c** Seed 2 results (s2δ). Purple line represents the significant FC link whose value is included in the correlation analysis. The correlation between mδ <−> s2δ FC and physical activity is shown on the right

Applying NBS methodology independently for each frequency band, three significant clusters emerged, located mainly on the left temporal lobe (see Table 2). Using TPA, we found two significant main (m) clusters, one in the theta band (mθTPA, Fig. 1) and one in the delta band (mδTPA, Fig. 2a). In the case of MVPA, only one cluster in the delta band showed a significant correlation with FC-st (mδMVPA). Since mδTPA and mδMVPA overlapped to a great degree, only mδTPA is depicted in Fig. 2. In the three clusters, FC-st negatively correlated with PA; thus, higher levels of PA were associated with lower cluster FC-st. In addition, the correlation between PA and FC-st in both delta band clusters remained significant when looking at the *APOE* ε4 carrier and non-carrier groups separately. The cluster in the theta band was significant among *APOE* ε4 carriers; however, within the *APOE* ε4 non-carriers, the relationship was not significant. To further assess these potential interaction effects, we conducted a moderation analysis. We observed a significant moderation effect of *APOE* genotype for the mθTPA cluster (*p* = 0.044) while no significant effect was observed for mδTPA (*p* = 0.13) nor mδMVPA (*p* = 0.055).

**Table 2:** Main clusters presenting decreased FC-st at higher PA levels

Cluster	mθTPA	mδTPA	mδMVPA
ROIs	Left amygdala (100%)	Left amygdala (100%)	Left fusiform gyrus (13.3%)
	Left hippocampus (20%)	Left fusiform gyrus (13.3%)	Left inferior frontal gyrus, orbital (33.3%)
	Left inferior frontal gyrus, orbital (8.3%)	Left inferior frontal gyrus, orbital (41.7%)	Left inferior temporal gyrus (16.7%)
	Left inferior temporal gyrus (4.2%)	Left inferior frontal gyrus, opercular (28.6%)	Left insula (50%)
	Left insula (50%)	Left inferior temporal gyrus (12.5%)	Left middle temporal gyrus (13.6%)
	Left middle temporal gyrus (15.9%)	Left insula (71.4%)	Left temporal pole, middle temporal gyrus (85.7%)
	Left postcentral gyrus (2.9%)	Left middle temporal gyrus (27.3%)	Left temporal pole, superior temporal gyrus (70%)
	Left superior temporal gyrus (50%)	Left parahippocampus (25%)	Left superior temporal gyrus (15%)
	Left temporal pole, middle temporal gyrus (28.6%)	Left postcentral gyrus (11.8%)	
	Left temporal pole, superior temporal gyrus (40%)	Left rolandic operculum (40%)	
		Left superior temporal gyrus (45%)	
		Left temporal pole, middle temporal gyrus (85.7%)	
		Left temporal pole, superior temporal gyrus (70%)	

Total physical activity (TPA) and daily average of moderate to vigorous physical activity (MVPA) correlated with functional connectivity strength (FC-st) in three main clusters. The list of regions of interest (ROIs) upon each significant main cluster fall is shown (in alphabetical order). The percentage of each ROI captured by each cluster is presented in brackets

Within the current study, greater levels of PA are associated with lower left temporal functional connectivity in both *APOE* ε4 carriers and non-carriers. Our findings are relevant to AD risk, as prodromal AD is usually characterized, in low frequency bands, as a stage of temporal lobe hyperexcitability [18–22].

### Decreased left temporal FC-st is mainly driven by reduced temporo-occipital and temporo-frontal FC

Decreased cluster FC-st indicates that the oscillatory activity (within a given frequency band) of the cluster regions is less synchronously paired with activity from all across the brain. However, in order to more specifically identify which connections drove such an effect, we performed a seed-based analysis. In this seed-based correlation analysis, we identified the specific connections (secondary clusters) of each of the main clusters with the rest of the brain that were significantly modulated by PA. We found two significant secondary (s) clusters for each main cluster in the delta band (s1δTPA and s2δTPA for mδTPA, Fig. 2b, c, respectively, and s1δMVPA and s2δMVPA for mδMVPA). The detailed list of areas belonging to these clusters is shown in Table 3. This result was significant among the whole sample and both APOE ε4 carriage subgroups. No significant secondary clusters emerged for mθTPA.

**Table 3:** Seed-based analyses

Seed	mδTPA		mδMVPA	
Cluster	Anterior (s1δTPA)	Posterior (s2δTPA)	Anterior (s1δMVPA)	Posterior (s2δMVPA)
ROIs	Left cingulate gyrus, anterior part (10.5%)	Left calcarine fissure and surrounding cortex (20%)	Left middle frontal gyrus (11.8%)	Left calcarine fissure and surrounding cortex (20%)
			Left precentral gyrus (9.4%)	Left cuneus (9.1%)
	Left cingulate gyrus, medial part (50%)	Left lingual gyrus (7.1%)	Left superior frontal gyrus (18.5%)	Left lingual gyrus (7.1%)
	Left middle frontal gyrus (17.6%)	Right calcarine fissure and surrounding cortex (50%)	Left supplementary motor area (4.2%)	Right calcarine fissure and surrounding cortex (16.7%)
	Left paracentral lobule (33.3%)			
				Right lingual gyrus (22.2%)
	Left precentral gyrus (15.6%)	Right cuneus (30.8%)		
	Left superior frontal gyrus (11.1%)	Right lingual gyrus (33.3%)		
	Left supplementary motor area (50%)	Right middle occipital lobe (17.6%)		
	Left superior frontal gyrus, medial (5.9%)	Right middle temporal gyrus (5.4%)		

Each main cluster (mθTPA, mδTPA, and mδMVPA) whose functional connectivity strength (FC-st) was significantly correlated with PA was used as a seed in a seed-based analysis. Clusters in the delta band (mδTPA and mδMVPA) presented lower FC to two extra clusters each (s1δTPA, s2δTPA, s1δMVPA, and s2δMVPA). The regions of interest (ROIs) that are comprised in each additional cluster are presented. The percentage of each ROI captured by each cluster is presented in brackets

### Lower temporal lobe FC is differently associated with cognitive function and brain structure in APOE ε4 carriers and non-carriers

Once we had described how greater levels of PA related to a distinctive FC-st profile, we aimed to characterize the relationship between this profile and parameters of brain health in order to better understand our results. Significant correlations were quite consistent across clusters. In the whole sample, most AD markers negatively correlated with FC-st, so that lower FC-st values were associated with healthier scores over different domains. Additionally, structural measures (total gray matter, hippocampus, precuneus, and amygdala volumes, as well as parahippocampal fasciculus fractional anysotropy) were significantly negatively associated with FC-st among *APOE* ε4 carriers. Anxiety levels also significantly correlated with FC-st in this group, so that lower FC-st was associated with lower anxiety levels. In contrast, only a few significant correlations were found in *APOE* ε4 non-carriers, all of them related to cognition (working and episodic memory). The complete set of correlation results can be found in Table 4.

**Table 4:** Correlation analyses

	Whole sample						Carriers						Non-carriers					
	mθTPA		mδTPA		mδMVPA		mθTPA		mδTPA		mδMVPA		mθTPA		mδTPA		mδMVPA	
	Rho	*p*	Rho	*p*	Rho	*p*	Rho	*p*	Rho	*p*	Rho	*p*	Rho	*p*	Rho	*p*	Rho	*p*
Forward digits	− 0.20	0.03	− 0.18	0.061	− 0.20	0.04	− 0.30	0.09	− 0.08	0.66	− 0.04	0.84	− 0.17	0.14	− 0.23	0.05	− 0.28	**0.01**
Reverse digits	− 0.23	**0.02**	− 0.21	**0.03**	− 0.22	**0.03**	− 0.29	0.11	− 0.23	0.20	− 0.22	0.23	− 0.23	0.05	− 0.22	0.06	− 0.22	0.06
Logical mem. units	− 0.00	0.96	− 0.00	0.99	− 0.03	0.78	− 0.11	0.54	− 0.16	0.40	− 0.12	0.51	0.05	0.65	0.09	0.47	0.06	0.63
Logical mem. gist	− 0.18	0.07	− 0.27	**0.01**	− 0.23	**0.02**	− 0.09	0.62	− 0.13	0.51	− 0.09	0.66	− 0.20	0.10	− 0.34	**0.01**	− 0.30	**0.01**
Anxiety	0.29	**< 0.01**	0.22	0.03	0.2	0.05	0.46	**0.01**	0.28	0.15	0.29	0.13	0.20	0.09	0.19	0.13	0.16	0.18
Depression	0.08	0.423	0.08	0.43	0.079	0.44	− 0.04	0.84	− 0.06	0.72	− 0.11	0.54	0.14	0.25	0.17	0.17	0.20	0.10
Total GM	− 0.27	**0.01**	− 0.29	**< 0.01**	− 0.29	**< 0.01**	− 0.39	0.03	− 0.44	**0.01**	− 0.43	**0.02**	− 0.23	0.05	− 0.23	0.05	− 0.23	0.05
Amygdala	− 0.28	**< 0.01**	− 0.27	**< 0.01**	− 0.27	**< 0.01**	− 0.33	0.07	− 0.41	**0.02**	− 0.39	**0.03**	− 0.25	0.03	− 0.21	0.08	− 0.21	0.07
Hippocampus	− 0.26	**0.01**	− 0.27	**< 0.01**	− 0.28	**< 0.01**	− 0.50	**< 0.01**	− 0.47	**0.01**	− 0.42	**0.02**	− 0.16	0.18	− 0.17	0.15	− 0.20	0.08
Precuneus	− 0.14	0.15	− 0.20	0.04	− 0.21	**0.03**	− 0.15	0.41	− 0.42	**0.02**	− 0.42	**0.02**	− 0.11	0.35	− 0.10	0.42	− 0.10	0.41
Global FA	− 0.03	0.72	− 0.08	0.43	− 0.04	0.69	− 0.18	0.33	− 0.16	0.38	− 0.11	0.56	− 0.00	0.97	− 0.04	0.73	− 0.00	0.98
Uncinate	− 0.24	**0.02**	− 0.18	0.07	− 0.14	0.16	− 0.61	**< 0.01**	− 0.44	**0.01**	− 0.39	**0.03**	− 0.07	0.58	− 0.07	0.59	− 0.01	0.91
Parahippocampus	− 0.00	0.96	− 0.07	0.49	− 0.03	0.76	− 0.17	0.37	− 0.30	0.10	− 0.22	0.23	0.05	0.68	0.03	0.77	0.07	0.55

Results for Spearman’s correlation analyses between mean FC-st of each main cluster (mθTPA, mδTPA, and mδMVPA) and a series of AD markers (rho and *p* values) are shown. The list of variables includes the following: working memory (Forward and Reverse Digit Span—forward and reverse digits); episodic memory (Logical Memory II—delayed recall; for units and gist—logical mem. units and gist); anxiety (Goldberg’s test); depression (Geriatric Depression Scale); total gray matter volume (GM, in cubic millimeter); hippocampus, amygdala, and precuneus volumes (left plus right, in cubic millimeter); global fractional anisotropy (FA); and uncinate and parahippocampal fasciculus fractional anisotropy (left and right weighted arithmetic mean). Outcomes that were significant for α < 0.05 and FDR *q* = 0.1 are bolded

## Discussion

**Fig. 3:**
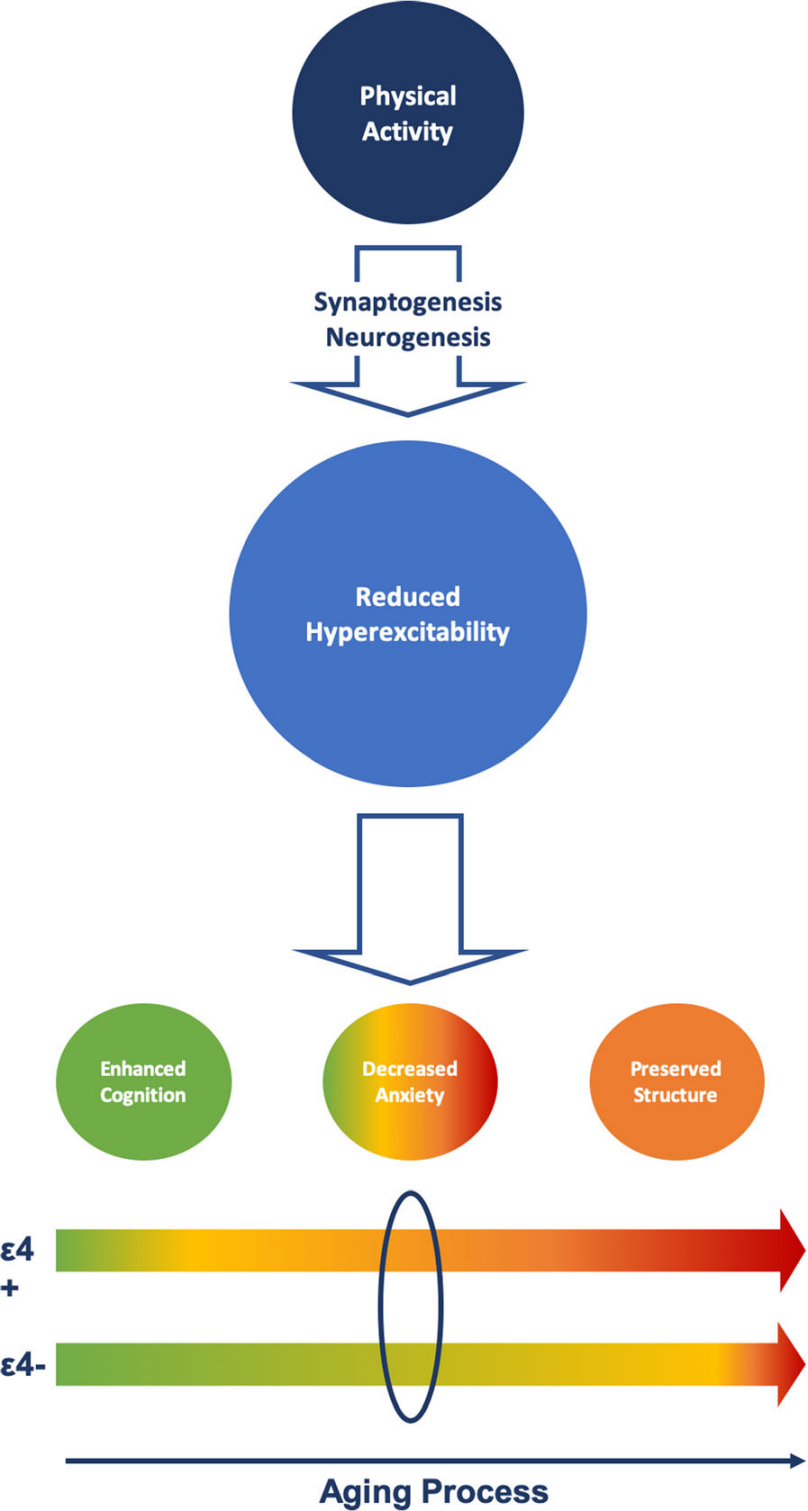
Proposed mechanism for physical activity-induced beneficial effects on brain health in *APOE* ε4 carriers and non-carriers. Physical activity (PA) is associated with decreased functional connectivity (FC) both in *APOE* ε4 carriers and non-carriers. We propose that this relationship could be mediated by a PA-induced increase in neurogenesis and synaptogenesis. Such processes could in turn prevent the loss of inhibitory synapses that has been identified to cause hyperexcitability in temporal regions in prodromal Alzheimer’s disease (AD). Interestingly, this decrease in FC manifests differently according to AD genetic risk. In ε4 carriers, this profile is linked to reduced anxiety and preserved brain structure. Conversely, in ε4 non-carriers, it is associated with enhanced cognition. One possibility behind this pattern of results could be that ε4 carriers were at higher risk of neuronal damage, which in normal aging would appear later. Therefore, at the specific time when we are taking these measurements, PA has more room to exert its beneficial effect on brain structure in ε4 carriers, while in non-carriers, at lower risk for neuropathological burden, it is associated with improved cognitive functioning. Hence, it remains plausible that at older ages, PA could also relate to greater structural integrity. However, we cannot rule out the possibility that PA affected ε4 carriers and non-carriers through different mechanisms

The purpose of the current study was to deepen our understanding of the role that *APOE* ε4 plays as a modulator in the relationship between PA and brain structure and function. The most relevant finding of the present work is that greater engagement in PA is related to lower left temporal FC, both in *APOE* ε4 carriers and non-carriers. Similar results were obtained with volumes of both total PA and PA at moderate to vigorous intensity. This FC profile was correlated with varying beneficial effects in AD-related features in both *APOE* ε4 carriers and non-carriers. However, these favorable associations differed according to AD genetic risk. More specifically, we found a relationship between region-specific decrease in FC-st and greater total GM volumes, greater integrity of the uncinate fasciculus, higher episodic and working memory scores, and reduced anxiety levels across the whole sample. In the *APOE* ε4 non-carriers only, network profile correlated with enhanced episodic and working memory, cognitive skills known to be affected early in the course of AD. In contrast, in *APOE* ε4 carriers, left temporal hypoconnectivity was associated with more preserved brain structure, particularly in areas that are more vulnerable to AD pathology (hippocampus, precuneus, amygdala, and uncinate tract). Figure 3 summarizes these results.

Our finding that PA is associated with reduced temporal lobe hypersynchrony in healthy older adults (even in individuals at greater genetic risk for AD) is noteworthy, considering that AD has been traditionally described as a disconnection syndrome [39]. However, recent evidence is building on the idea that preclinical AD is characterized by a dual neurophysiological profile. Through MEG, it has been discovered that in subjective memory decline and mild cognitive impairment, a state of hypersynchrony precedes the well-known phase of hypoconnectivity [18–22]. A closer look at these individuals’ brain microstructure provides a plausible explanation for these chronological changes. At the very early silent stages of AD pathology, inhibitory neurons are lost, mainly in middle temporal regions [13, 14]. Such loss of inhibitory synapses leads to a state of brain hyperexcitability and hypersynchrony, which can be tracked through MEG [15]. Sustained hyperactivation elicits neurotoxic effects and increased release of amyloid (which ultimately lead to neuronal damage [16, 17]). As a result, extensive brain atrophy and generalized hypoconnectivity are evident features by the time AD clinical symptomatology arises [40].

It is interesting that it was the left temporal lobe which exhibited significant results, since decreased synchronization in AD, already at the dementia stage, seems to mainly affect the left hemisphere [41]. On the other hand, both normal aging and AD brain activity presents a marked “slowness,” this is an increase in power in low frequency bands (delta and theta) [42]. In fact, increases in delta connectivity had already been described as a pathological sign in other clinical conditions, as well as decreases upon cognitive recovery [43]. It is possible then that since low frequency rhythms are more associated with brain neurophysiological health, PA could exert its beneficial effect by affecting these rhythms specifically. Conversely, although there are not many functional connectivity studies that could provide an explanation on why physical activity affects those frequency bands in particular, power spectrum studies suggest that during an acute bout of exercise, activity in the theta band is enhanced. Such increase in theta power is believed to serve a cognitive function, as physical activity is evolutionary associated with increases in cognitive demands [44]. This effect is usually reversed immediately after the physical activity bout ceases [45, 46]. This phenomenon could be related to the diminished FC-st within the theta band that we find in active individuals at rest.

In an attempt to understand the meaning of our FC results, we also studied the association between the observed brain activity patterns and cognitive/emotional functioning, brain volumes, and white matter integrity: within these analyses, differences between *APOE* ε4 carriers and non-carriers are evident. Previous literature offers mixed results with regard to whether *APOE* ε4 carriers or non-carriers gain the greatest benefit from PA engagement. This inconsistent literature may be a result of the utilization of varying PA measurements (questionnaires, fitness measures, and PA interventions) and different outcomes (AD risk, cognitive scores, or brain activity/structure), conducted in samples of different characteristics (in terms of age, AD risk, and cognitive status). In the current study, we examined the effect of objectively measured PA on a wide range of AD markers employing a sample of cognitively healthy *APOE* ε4 carriers and non-carriers properly matched on an extensive list of potential confounders.

While measures of different brain volumes correlated with FC-st in the ε4 carrier group, these associations did not exist in ε4 non-carriers. The same pattern arose when looking at the integrity of the uncinate tract. It is important to highlight that these are all brain structures that are particularly vulnerable to AD pathology. Since this is a sample of cognitively healthy participants, our results could be better understood if we consider that individuals at increased genetic risk already present greater variability in GM and WM state of preservation. Therefore, we could assume that there is more room for PA to counterbalance early neuropathological signs within this group. This is consistent with previous studies that demonstrate the effect of PA on brain pathology is most predominant among ε4 carriers [7, 47, 48].

Contrary to our aforementioned findings within *APOE* ε4 carriers, we observed a relationship between decreased FC and greater scores in specific measures of episodic and working memory, but only among *APOE* ε4 non-carriers. Most studies investigating the relationship between AD incidence and PA have concluded that only *APOE* ε4 non-carriers benefit from reduced AD risk at greater levels of PA [9, 49, 50], although there are some exceptions [51, 52]. Presently, AD diagnosis is based on clinical progression and cognitive status. Therefore, our finding that the profile of FC associated with PA only predicts cognitive functioning among *APOE* ε4 non-carriers is somewhat consistent with previous research.

Finally, across the whole sample and *APOE* ε4 carriers, we found a positive correlation between greater FC-st and higher anxiety. In previous studies, anxiety has been identified as a marker of conversion from preclinical AD to AD [53, 54]. In addition, higher levels of anxiety are related to greater temporal lobe atrophy [55]. Hence, our results demonstrate that PA-associated reduced FC-st is also associated with lower anxiety levels and greater temporal lobe volumes.

PA has been widely studied as a protective ally against AD. Our study sheds light on the potential mechanisms through which PA could exert its action. According to the neurogenic reserve hypothesis, throughout evolution acute bouts of PA were linked to an increased likelihood of a potential cognitive challenge [56, 57]. As hunter-gatherers, going through long distances relied on improved spatial orientation, memory, and executive functions. Locomotion would signal the brain such increase in cognitive demands. In response, the reserve of neuronal precursor cells would grow. In the presence of cognitive stimulation, new neurons would maturate, differentiate, and migrate. Multiple sources of evidence support the postulate that PA promotes synaptogenesis and neurogenesis, mainly within the hippocampal network [58, 59]. Newborn granule cells do not produce hyperactivation but rather present sparse activity during learning [60]. Such mechanisms could explain the decreased temporal FC profile that we detect in older adults who regularly engage in greater levels of PA. But most importantly, they could explain why PA is one the most relevant modifiable protective factor against AD. Indeed, Raicheln and Alexander hypothesize that 2 million years ago, PA was able to counterbalance the detrimental effects of *APOE* ε4, when our ancestors carried two copies of this risk allele [61]. However, although in this study we report that the association between PA and FC-st is associated to beneficial effects in both ε4 carriers and non-carriers, it remains possible that the underlying mechanisms differ based on AD genetic risk.

The results observed in the current study provide a more comprehensive picture of the relationship between PA, *APOE*, and AD pathology, compared with previously conducted studies. Our findings help strengthen the understanding of the complex dynamics that underpin the varying outcomes observed in previous studies. Although the age range in this sample was fairly broad, the mean age was still quite low to study the effects of advanced aging or prodromal AD. Therefore, it is possible that we missed out on certain effects that might only appear at later stages of life. Follow-up studies are required to determine how the FC profiles identified within this study are associated with pathological progression and cognitive change among, at-present, cognitively healthy older adults. Also, future studies should include a group of *APOE* ε4 homozygotes, since our sample size did not allow us to incorporate that comparison. Additionally, it would be interesting to see how diverse PA parameters, such as the type of activity or frequency of practice, affect certain markers of the disease. This information could be useful in the elaboration of lifestyle guidelines aiming to promote brain health. Unfortunately, such measures were not available from this cohort. In addition, it would be useful to include specific AD biomarkers to better characterize brain health in individuals at risk, instead of relying solely in genetic risk factors to identify individuals at greater risk of developing dementia.

## Conclusions

Altogether, our study offers novel insights into this field, clarifying some of the specific mechanisms that underlie the beneficial effect of PA in *APOE* ε4 carriers and non-carriers. It enables the integration of previous publications and leads the way to future findings. Previous literature offered apparently inconsistent results, but our study suggests that the specific brain health outcomes considered could be differently affected by PA in ε4 carriers and non-carriers. Nevertheless, we were able to describe an association between PA and decreased temporal lobe hypersynchrony across the whole sample that highlights the need to design new policies that foster PA among older adults, including those more vulnerable to develop AD.

## Data Availability

The datasets used and/or analyzed during the current study are available from the corresponding author on reasonable request.

## Abbreviations

AAL: Automated Anatomical Labeling atlas
AD: Alzheimer’s disease
APOE: Apolipoprotein E
DWI: Diffusion-weighted images
EDTA: Ethylenediaminetetraacetic acid
FA: Fractional anisotropy
FC: Functional connectivity
FC-st: Functional connectivity strength
FDR: False discovery rate
GM: Gray matter
mθTPA: Main cluster found in the theta band using TPA
mδTPA: Main cluster found in the delta band using TPA
mδMVPA: Main cluster found in the delta band using MVPA
MEG: Magnetoencephalography
MNI: Montreal Neurological Institute
MVPA: Moderate to vigorous physical activity
NBS: Network-based statistics
PA: Physical activity
PLV: Phase locking value
ROI: Region of interest
TPA: Total physical activity
s1δTPA and s2δTPA: Secondary clusters associated with mδTPA
s1δMVPA and s2δMVPA: Secondary clusters associated with mδMVPA
SNP: Single nucleotide polymorphism
WAIS-IV: Weschler Adult Intelligence Scale IV
